# Regional variations in Paneth cell antimicrobial peptide expression along the mouse intestinal tract

**DOI:** 10.1186/1471-2172-9-37

**Published:** 2008-07-17

**Authors:** Jenny Karlsson, Katrin Pütsep, Hiutung Chu, Robert J Kays, Charles L Bevins, Mats Andersson

**Affiliations:** 1 Department of Microbiology, Tumor and Cell Biology, Karolinska Institutet, Stockholm, Sweden; 2 Department of Microbiology and Immunology, School of Medicine, University of California at Davis, Davis, CA, USA

## Abstract

**Background:**

Enteric antimicrobial peptides secreted from Paneth cells, including α-defensins (in mice named cryptdins), are key effector molecules of innate immunity in the small intestine. The importance of Paneth cells α-defensins emerged from studies of enteric bacterial infection in genetically modified mice, as well as from recent studies linking reduced levels of these α-defensins to Crohn's disease localized to the ileum. However, analysis of expression of Paneth cell α-defensins is incomplete. We therefore performed a comprehensive evaluation of the distribution of antimicrobial molecules along the mouse small intestinal tract to identify potential variations in regional expression.

**Results:**

In conventionally reared mice, the repertoire of Paneth cell antimicrobials differs between duodenum and ileum. In contrast to the uniform expression of most Paneth cell antimicrobials, both cryptdin 4 and cryptdin-related sequences (CRS) 4C peptides were expressed at progressively increasing amounts (10^1^- and 10^4^-fold, respectively) comparing duodenum and ileum. In tissues other than the small intestine, expression of CRS peptides was noted in thymus and caecum. Most Paneth cell products were also produced in the small intestine of germ-free mice at levels similar to those in controls, however CRS4C and RegIIIγ had reduced levels in the former (3- and 8-fold, respectively). No significant changes in expression levels of Paneth cell antimicrobial peptides was observed after oral challenge with either *Salmonella enterica *serovar typhimurium or *Listeria monocytogenes*, supporting current notions on the constitutive nature of this defensive system.

**Conclusion:**

The repertoire of antimicrobial peptides changes along the small intestinal tract, and a subset of these molecules are up-regulated upon colonization, but not in response to enteric bacterial pathogens. The changes detected upon colonization suggest that Paneth cell antimicrobial peptides may play an important role in commensal microbial homeostasis, in addition to their proposed role in protection against infection. In addition, the differential expression of CRS4C along the small intestine suggests mechanisms of regulation that are distinct from other Paneth cell derived antimicrobial peptides.

## Background

The gastrointestinal epithelium is continuously exposed to high numbers of bacteria, including the resident microbiota as well as to foreign bacteria. Despite this high bacterial exposure, infections are rare, which suggests highly efficient mechanisms both to protect the host and to maintain a host-microbe balance. Protection in the small intestine is attributed, in part, to a mucosal barrier composed of mucins and secreted antimicrobial components from Paneth cells. Paneth cells are highly differentiated, long-lived [1] secretory cells that are located at the base of crypts of Liberkühn. Paneth cell granules are released by neural stimulation or in response to luminal bacteria or bacterial compounds [2]. Many important antibacterial components, such as α-defensins (cryptdins in mice), lysozyme, phospholipase A2 and the C-type lectin RegIIIγ are produced at high levels and stored in Paneth cell granules [3,4]. Humans have only two α-defensins [5] whereas mice have many cryptdins and cryptdin-related sequence (CRS) peptides expressed in their Paneth cells [3,6]. Most of the mouse cryptdins belong to the highly similar cryptdin 1 group, with the exceptions being cryptdins 4 and 5 [3]. The cell specific regulation of Paneth cell α-defensins has partly been unravelled with the recent identification of the transcription factor TCF-4 being a key protein [7,8].

The CRS peptide family in mice was initially described at the mRNA level based on its similarities to the pro-sequence of cryptdins [6,9,10], but the processed peptide shares no similarities with cryptdins. Mature CRS peptides are divided into two groups; CRS1C containing 11 cysteines and CRS4C with 9 (10) cysteines [9,11]. We have shown previously that CRS4C peptides have antimicrobial activity against both Gram-positive and Gram-negative bacteria [11]. The antimicrobial spectra of these peptides are broadened by the fact that they form both homo- and heterodimers, which have distinct but overlapping antimicrobial activities [11].

The importance of the small intestinal antimicrobial peptides has been highlighted in both human and mice. Recently, a reduction of Paneth cell α-defensins was implicated as a key contributor to the innate immune dysfunction seen in Crohn's ileitis [12]. In addition, mice with deficient processing of cryptdins are more sensitive to oral infection by *Salmonella enterica *serovar typhimurium [13], whereas transgenic mice with one additional human α-defensin expressed in Paneth cells are more resistant to Salmonella [14]. Together, these findings support the hypothesis that Paneth cell defensins are key effector molecules of innate immunity in the intestinal tract, but many questions remain to be solved regarding their expression and regulation.

The data presented here demonstrates that Paneth cells along the longitudinal axis of the small intestine are heterogeneous with respect to their expression of antimicrobial peptides. The most significant heterogeneity was detected for the CRS peptide CRS4C, with a 10^4^-fold increased expression in the ileum compared to the duodenum. Consistent with previous studies, the expression of Paneth cell antimicrobials varied little in response to bacterial pathogens, suggesting that this defense system is an ever-present arm of innate immunity. An evaluation of the levels in germ-free versus conventionally housed mice revealed that the relative distribution of some Paneth cell antimicrobials was influenced by bacterial colonization. Together these data provide a foundation to help in the design and interpretation of mouse models of enteric infection and inflammation.

## Results

### Variant CRS sequences in FVB mice

Previous studies identified variant CRS transcript forms when comparing different mouse strains [6,9,11,15]. We therefore sought to investigate the transcripts derived from the small intestine of FVB mice using an anchored PCR strategy (for primers see Table 1). We identified ten different deduced CRS peptide sequences from 21 sequenced clones (Table 2). The sequences were compared from the amino terminus of the mature peptide (here defined as LQD) through to the stop codon. Four clones encoded CRS1C peptides and six encoded CRS4C peptides, Table 2. Both previously annotated sequences and novel sequences were found. In addition, we noted that three clones coded for the same active peptides, but had sequence variations in the 3'-non-coding region (data not shown). This suggests that the same active peptide can be encoded by several gene paralogs.

**Table 1:** Primers used for cloning of CRS peptides and for qRT-PCR assays

**Primers used for cloning of CRS peptides**		
**Product**	**Sense**	**Antisense**

**CRS**	5'aaggctctg(c/g)tcttcaagat3' 5'tg(c/g)tcttcaagatg(t/c)agccc3'	5'ccatcctaatacgactcactatagggc3'
**CRS4C-4**	5'gtgagtgctggactcagcc3'	5'gattgcatttgcagctcggg3'
**CRS4C-6**	5'cctaggaaagcctccccagt3'	5'ttttgtcatgcaattgcacc3'
		

**Primers used for qRT-PCR assays**		

**CRS1C**	5'caccacccaagctccaaatacacag3'	5'atcgtgaggaccaaaagcaaatgg3'
**CRS4C**	5'gcatggaatctgggtcaagataac3'	5'agaaggaagagcaatcaaggctaag3'
**Crypt1**	5'tcaagaggctgcaaaggaagagaac3'	5'tggtctccatgttcagcgacagc3'
**Crypt4**	5'gctgtgtctatctcctttggaggc3'	5'cgtattccacaagtcccacgaac3'
**mLys**	5'ggctggctactatggagtcagcctg3'	5'gcattcacagctcttggggttttg3'
**pLys**	5'gccaaggtctaacaatcgttgtgagttg3'	5'cagtcagccagcttgacaccacg3'
**sPLA2**	5'aggattcccccaagatgccac3'	5'cagccgtttctgacaggagttctgg3'
**TCF-4**	5'cggctcactccacagctcaaag3'	5'cgagaatctggttgatggctgc3'
**NOD2**	5'cgacatctcccacagagttgtaatcc3'	5'ggcacctgaagttgacattttgc3'
**MMP7**	5'ttcaagagggttagttgggggactg3'	5'ttgtcaaagtgagcatctccgcc3'
**Reg3g**	5'cctcaggacatcttgtgtctgtgctc3'	5'tccacctctgttgggttcatagcc3'
**Crypt4 C57BL/6**	5'ccaggggaagatgaccaggctg3'	5'tgcagcgacgatttctacaaaggc3'

Note that two gene-specific sense primers were used to clone CRS cDNA.

**Table 2:** Deduced amino acid sequence of putative mature CRS peptides

	**Deduced amino acid sequence**	**mRNA accession number**	**Mouse strains**	**Reference**
**CRS1C sequences**				

**Novel CRS1C**				
CRS1C-4	LQDAAQRRFPWCRKCRVCQKCQVCQKCPVCPTCPQCPKQPLCEERQNKTAITTQAPNTQHKGC	EU760893	FVB	this report
CRS1C-5	LQDVAQRRFPWCRKCRVCQKCQVCQKCPVCPTCPLCPKQPLCEERQNKTAITTQAPNTQHKGC	EU760894	FVB	this report
CRS1C-6	LQDVAQRRFPWCRKCPVCQKCQVCQKCPVCPTCPQCPKLPLCKERQNKSAITTQAPNTQHKGC	EU760895	FVB	this report
				
**Annotated CRS1C**				
CRS1C	LQDVAQRRFPWCRKCRVCQKCQVCQKCPVCPTCPQCPKQPLCEERQNKTAITTQAPNTQHKGC	M33226	FVB, Outbred Swiss albino	[9]
CRS1C-1	LQDVAQRRFPWCRKCRVCQKCEVCQKCPVCPTCPQCPKQPLCKERQNKTAITTQAPNTHHKGC	XM_001006437	C57BL/6*	[15]
CRS1C-2	LQDVAQRRFLWCRKCPVCQKCQVCQKCPVCPTCPQCPKQPLCEERQNKTAITTQAPNTQHKGC	AY761183	C57BL/6	[15]
CRS1C-3	LQYVAQRRFPWCRKCPVCQKCQVCQKCPVCPTCPQCPKLPLCKERQNKSAITTQAPNTQHKGC	AY761184	C57BL/6	[15]
				

**CRS4C sequences**				

**Novel CRS4C-1/2/3**				
CRS4C-3f	LQDAALGWGRRCPWCPPCPNCRRCPRCPTCPSCNCNPK	EU760896	FVB	this report
				
**Annotated CRS4C-1/2/3**				
CRS4C-1a/e/f/h/j	LQDAALGWGRRCPQCPRCPSCPSCPRCPRCPRCKCNPK	M33227	FVB, Outbred Swiss albino, C3H/HeN, 129/SVJ	[9]
CRS4C-1b/d/g/i	LQDAAVGWGRRCPQCPRCPSCPSCPRCPRCPRCKCNPK	AJ564861	FVB, 129/SVJ, C3H/HeN	[6]
CRS4C-2/c/d	LQDAALGWGRRCPRCPPCPRCSWCPRCPTCPRCNCNPK	NM_007848	FVB, 129/SVJ, C3H/HeN	[6]
CRS4C-2b	LQDAALGWGRRCPRCPPCPRCSWCPGCPTCPRCNCNPK	AJ564871	C3H/HeN	[11]
CRS4C-3/a/b/e	LQDAALGWGRRCPRCPPCPNCRRCPRCPTCPSCNCNPK	AJ564873	FVB, 129/SVJ, C3H/HeN	[6]
CRS4C-3c	LQDAALGWSRRCPRCPPCPNCRRCPRCPTCPSCNCNPK	AJ564876	C3H/HeN	[11]
CRS4C-3d	LQDAALGWGRRCPRCPPCPNCRRCPRCPTCPRCNCNPK	AJ564877	FVB, C3H/HeN	[11]
				

**Related CRS**				

CRS4C-4	LQDAAVGMARPCPPCPSCPSCPWCPMCPRCPSCKCNPK	NM_007845	129/SVJ	[6]
CRS4C-5	LQDAAAIRRARRCPPCPSCLSCPWCPRCLRCPMCKCNPK	NM_007846	129/SVJ*	[6]
CRS4C-6	LQVSGLGKPPQCPKCPVCSKCPQCPQCPQCPGCPRCNCMTK	AY761185	C57BL/6	[15]

Underlined: Variations in amino acid sequence within the group of CRS1C and CRS4C, respectively. One example of mRNA accession number only. * = predicted from genome sequence, no mRNA found.

Both specific forward and reverse primers, as well as an anchored PCR strategy were used to screen for potential CRS4C-4 and CRS4C-6 transcripts in the small intestine (Table 1), but no sequences were detected in the FVB mice.

### CRS4C is stored in Paneth cell granules

**Figure 1:**
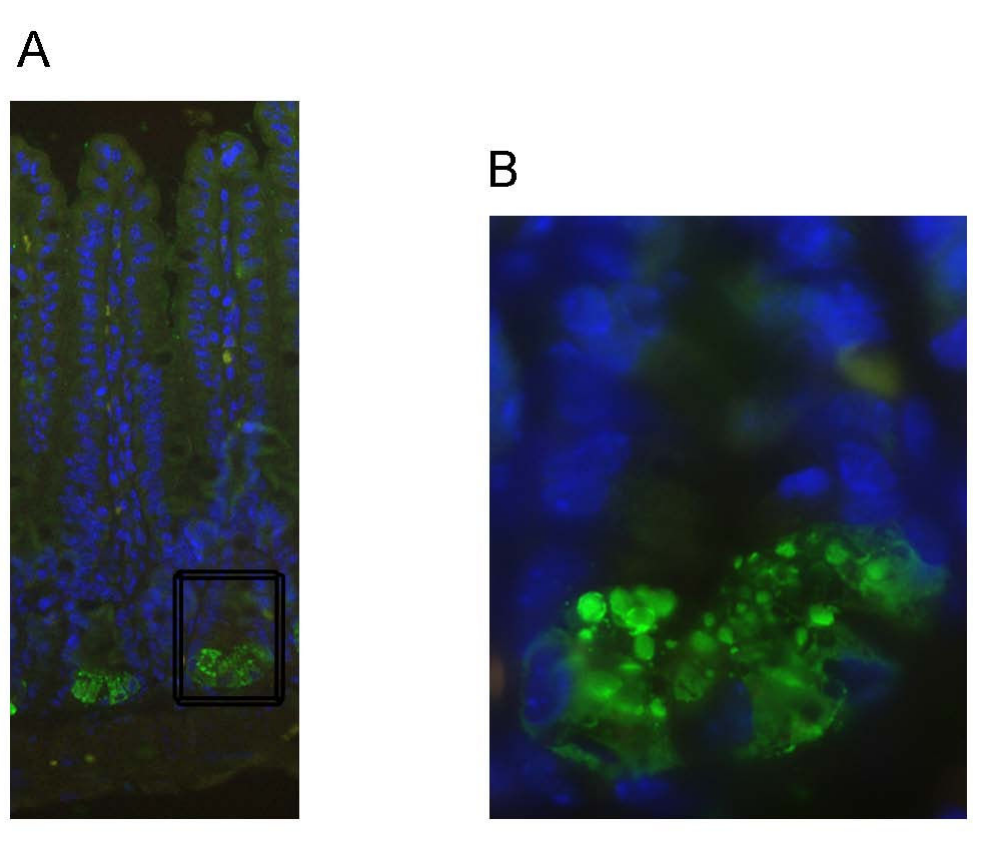
**Immunohistochemical localization of CRS4C in ileal part of the small intestine**. A) CRS4C (green) stains Paneth cell granules in crypts of Liberkühn of a C3H/HeN mouse. A higher magnification of the base of the crypt is shown in (B). Hoechst staining (blue) was used to visualize the nucleus.

We generated antibodies against CRS4C-3a in order to localize CRS peptides using immunohistochemistry. This antibody also recognizes synthetic CRS4C-1a, but the preimmune sera did not (data not shown). As shown in Figure 1, granules in Paneth cells from the ileum stained positive for CRS4C. Thus, CRS4C peptides are stored in granules similar to other characterized Paneth cell antimicrobials.

### Paneth cell antimicrobial differences in regional and developmental expression patterns

**Figure 2:**
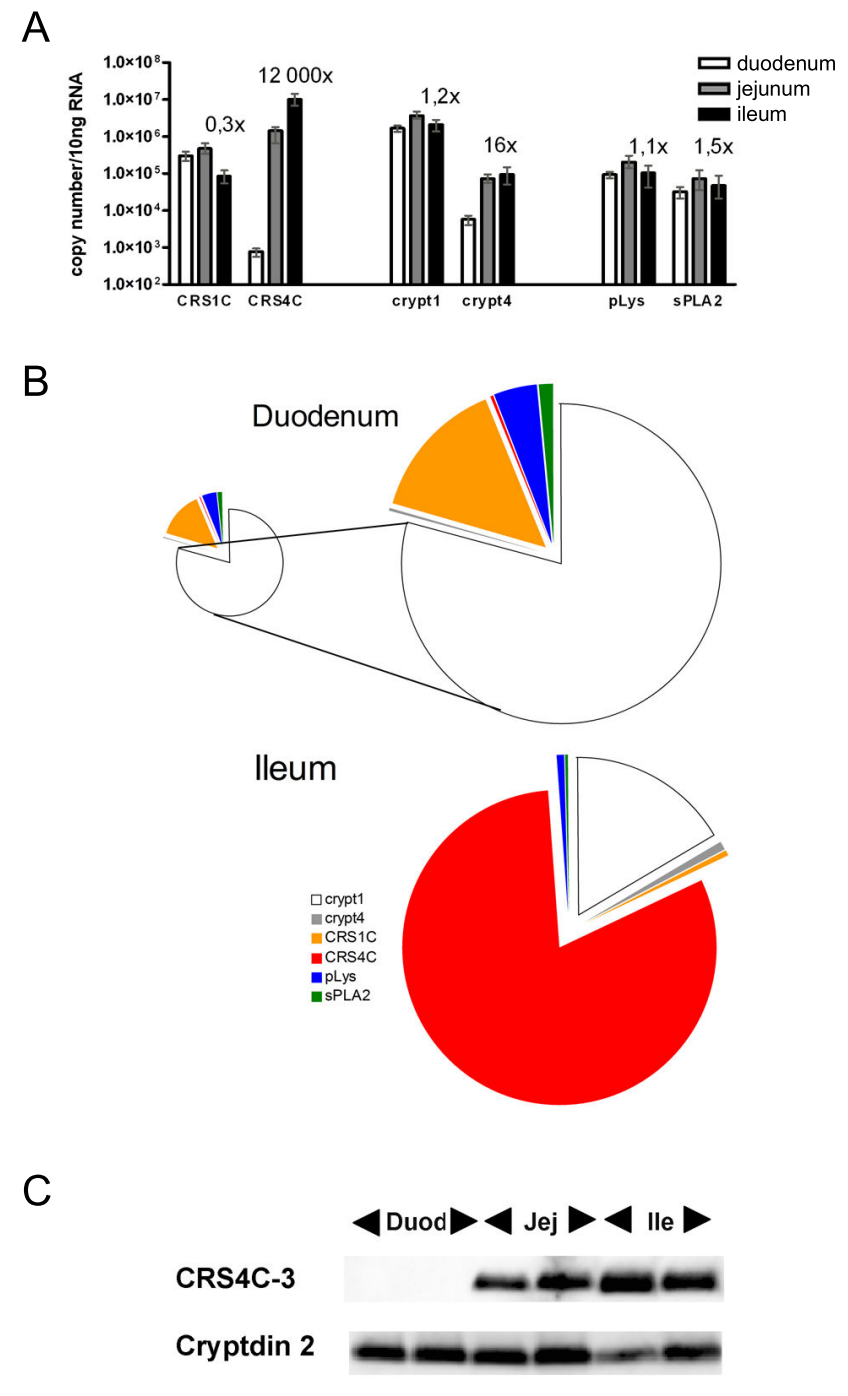
**Antimicrobial peptide expression along the mouse small intestine**. A) CRS, cryptdins, pLys, and sPLA2 mRNA expression in small intestine measured by qRT-PCR. Absolute mRNA copy numbers were determined in the duodenum, jejunum, and ileum. The "x" numbers denote relative copy numbers between ileum and duodenum. The results are based on four FVB mice, and are given as mean and range. B) Comparative distributions in duodenum and ileum. Of note is that duodenum has a total of 2 × 10^6 ^copies of Paneth cell products, while ileum has 18 × 10^6 ^copies (area of the smaller duodenal and the ileal pies are proportional to total mRNA copy counts of analyzed antimicrobials). Colours denote individual Paneth cell products. C) Western blot of extracts from two individual BALB/c mice was analyzed using antibodies to CRS4C-3 and cryptdin 2. Duod = duodenum, Jej = jenunum and Ile = ileum.

In order to compare the mRNA levels of Paneth cell derived antimicrobial molecules we used a quantitative real-time reverse transcriptase PCR (qRT-PCR) assay that measures the number of transcripts derived from 10 ng of RNA (primer sequences are found in Table 1). The normalization to the amount of total RNA renders comparison between different RNA sources and gene products possible. Measurements were made from segments (6 cm) of the duodenum, jejunum, and ileum tissue from each of four different adult FVB mice (from two different litters) in order to evaluate the spatial distribution of antimicrobial peptide expression. Consistently high expression of CRS1C was observed throughout the small intestine (Figure 2A). In contrast, the expression of CRS4C is region-specific, being low in duodenum and 12,000 times higher in ileum (Figure 2A).

We next quantified expression of other Paneth cell derived products using qRT-PCR (Figure 2A). The assay for cryptdin 1 also detects mRNA transcripts from cryptdin 2 and 3, but from here on is referred to as "cryptdin 1". Cryptdin 1, Paneth cell-lysozyme (pLys) and secretory phospholipase A2 (sPLA2) expression remained unchanged when comparing duodenum and ileum. Cryptdin 4 expression increased 16-fold from duodenum to ileum. The results for cryptdin 4 are in accordance with previous data [16,17], where the transcripts were measured using standard RT-PCR and immunohistochemistry, which makes quantitative comparisons with our results difficult. When comparing the mRNA levels of the different antimicrobial peptides in ileum, CRS4C had a five-fold higher expression than cryptdin 1 followed by CRS1C > cryptdin 4 > pLys and sPLA2 (Figure 2B). For the same analysis in the duodenum, the levels were, in decreasing order; cryptdin 1 > CRS1C > pLys > sPLA2 > cryptdin 4 ≈ CRS4C.

The protein levels of CRS4C and cryptdin 2 were determined in the duodenum, jejunum and ileum of two BALB/c mice by Western blot analysis (reported as microgram of peptide per gram of tissue, Figure 2C). The levels of CRS4C in the duodenum were below detection level, in jejunum were 39 μg/g and in the ileum were 54 μg/g. The level of cryptdin 2 was 74 μg/g for duodenum, 93 μg/g from jejunum and 66 μg/g from ileum.

**Figure 3:**
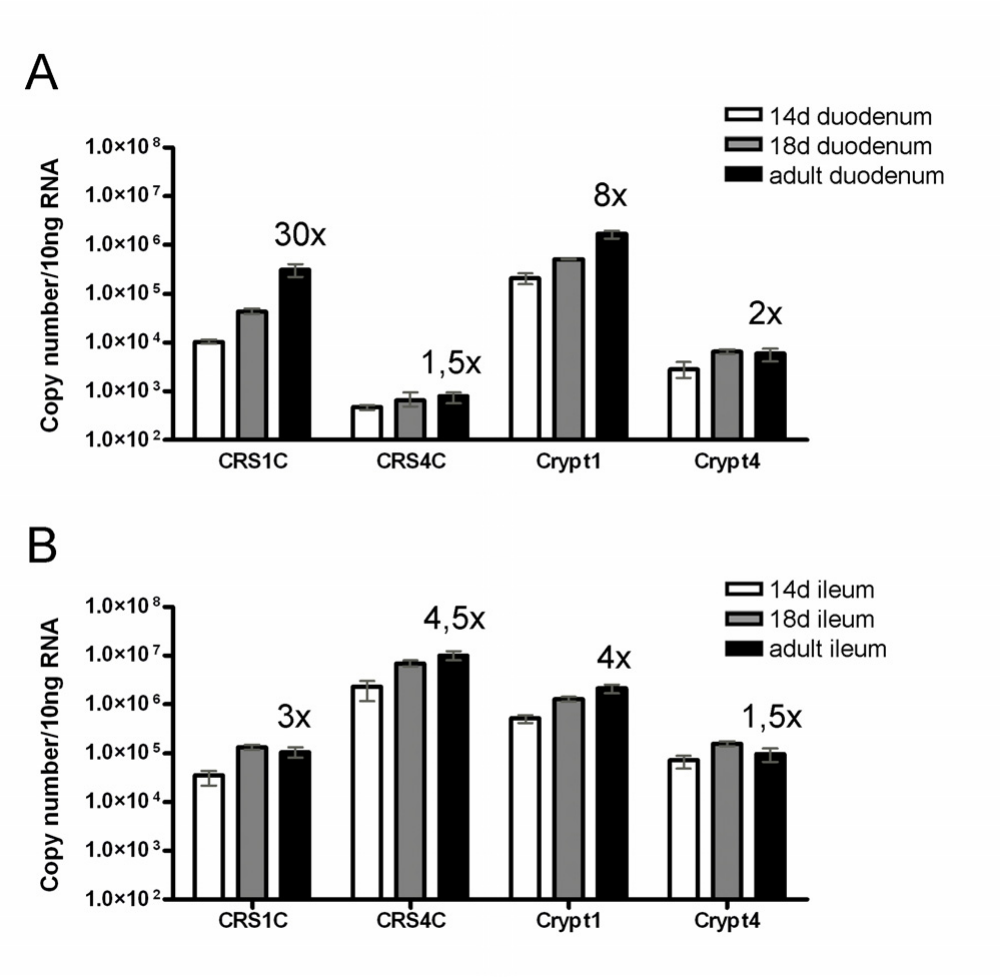
**mRNA expression of CRS and cryptdins during small intestinal development in A) duodenum, B) ileum**. Data is based on small intestinal tissue from three FVB mice in each group. Absolute mRNA copy numbers were determined in the duodenum and ileum at post-natal days 14 and 18 (preweaning) and adult mice. Means and range are depicted. The "x" numbers denote relative copy numbers measured by qRT-PCR between adult and 14 day old mice.

Antimicrobial peptide levels in duodenum and ileum from 14- and 18 day old mice were compared to that of adult levels (Figure 3). The mRNA for CRS1C and cryptdin 1 in duodenum was 30- and 8-fold higher,  respectively, in adult compared to day 14 tissue (Figure 3A). CRS4C and cryptdin 4 did not display such increase in duodenum, and remained at a constant low level during development. In the ileum, however, CRS4C and cryptdin 4, displayed higher expression in  adult compared to day 14 tissue, which was similar to cryptdin 1 and CRS1C (Figure 3B).

### Antimicrobial peptide expression in other tissues

We isolated RNA and measured transcript numbers from different tissues of FVB mice in order to identify potential tissues, apart from small intestine, where CRS and other antimicrobial molecules might be expressed. CRS and cryptdins were also detected in caecum and thymus (Table 3). The expression of CRS4C mRNA in both caecum and thymus was lower than in jejunum but higher than in duodenum. None of the cryptdins or CRS peptides were detected at appreciable levels in other tissues. Notably, the expression of cryptdins and CRS was not detected in other immune related sites such as bone marrow or spleen. The level of CRS protein expression was below the detection limit for Western blot (less than 3 μg/g tissue) and immunohistochemistry in caecum and thymus (data not shown).

**Table 3:** Distribution of antimicrobial peptide expression in different mouse tissues measured by qRT-PCR

	**CRS1C**	**CRS4C**	**Crypt1**	**Crypt4**	**sPLA2**	**pLys**	**mLys**	**GAPDH**
**tongue**	*	*	*	*	*	318	7,090	67,100
**trachea**	*	359	*	*	*	13,000	317,000	22,100
**esophagus**	*	*	*	*	*	438	29,900	101,000
**cardiac stomach**	*	882	*	*	*	*	4,730	16,000
**body stomach**	*	1,570	*	*	*	*	3,040	43,200
**pyloric stomach**	166	111	1,530	*	1,920	*	5,640	64,600
**caecum**	210	240,000	29,100	2,630	10,100	3,830	49,400	162,000
**ascending colon**	*	262	151	*	2,730	339	34,800	142,000
**transcending colon**	*	*	*	*	12,600	287	25,200	135,000
**descending colon**	*	*	*	*	9,680	400	34,000	191,000
**rectum**	*	*	*	*	7,010	445	89,100	72,300
								
**thymus**	1,950	19,100	622	224	*	1,820	265,000	48,300
**lung**	*	101	*	*	*	245,000	1,050,000	29,800
**bone marrow**	*	*	*	*	*	*	1,080,000	89,000
**skin**	*	625	*	*	*	*	542	*
**liver**	*	205	*	*	*	*	24,800	50,100
**salivary gland**	*	*	*	*	*	144	13,600	31,300
**heart**	*	*	*	*	*	152	21,400	202,000
**kidney**	*	*	*	*	*	*	5,610	190,000
**spleen**	115	393	*	*	*	179	114,000	38,200

Results are based on pooled tissues from 4–20 FVB mice. Caecum and thymus showed highest expression of cryptdins and CRS peptides. * = <100 copies/10 ng RNA. Three significant digits displayed.

### Impact of germ-free condition and bacterial infection on levels of Paneth cell antimicrobials

**Figure 4:**
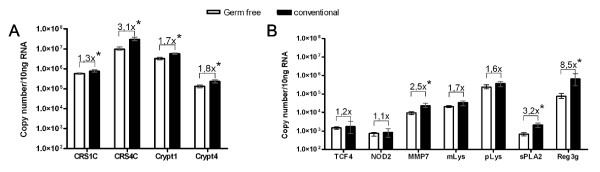
**Innate defense molecules expressed in small intestine of germ-free and conventional mice**. A) CRS and cryptdins, B) other mRNA transcripts detected in whole small intestine. Means and range are depicted. Numbers denotes relative copy numbers between small intestine of germ-free and conventional NMRI/KI mice. Four mice in each group. * denotes P < 0,03.

Germ-free NMRI/KI mice were used to assess the role of the resident microbiota on CRS peptide expression. RNA was isolated from whole small intestine of germ-free as well as conventional mice and transcript copy numbers were compared using qRT-PCR (Figure 4). The expression levels represent two categories, less than three-fold higher expression (CRS1C, cryptdin 1, cryptdin 4, NOD2, MMP7, mLys and pLys) and more than three-fold higher levels (CRS4C, sPLA2 and RegIIIγ). The higher expression of CRS4C, sPLA2 and RegIIIγ in conventionally reared mice could not be explained by TCF-4 expression since the levels of TCF-4 remained unchanged. This demonstrates that the normal flora influences the expression of a subset of Paneth cell peptides and that transcription factors other than TCF-4 likely contribute to this effect.

**Figure 5:**
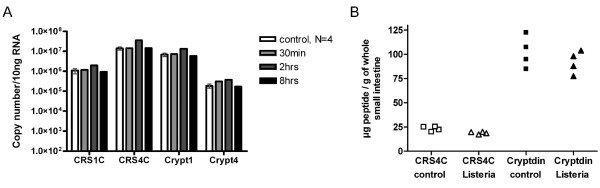
**Whole small intestinal expression of cryptdins and CRS peptides after infection**. A) CRS and cryptdins measured by qRT-PCR after Salmonella infection of C3H/HeN mice at different time points. B) CRS peptides and cryptdins measured by Western blot from four individual BALB/c after three days of Listeria infection (denoted by triangles). Four mice receiving PBS were used for comparison (squares).

Mice were intragastrically infected with *S. typhimurium *serovar typhimurium and whole small intestine was analyzed at different time points up to eight hours post infection. With qRT-PCR we did not observe any significant changes in transcript levels (Figure 5A). Three days after oral infection with *L. monocytogenes*, small intestine was analyzed. No changes in cryptdin 2 or CRS4C were detected using Western blot analysis (Figure 5B).

### CRS peptide levels vary in different mouse strains

A comparison of the CRS repertoire identified here in FVB mice to the previously reported collection of enteric CRS4C sequences in C3H/HeN mice [11] suggested a significant strain-to-strain variation, which we evaluated further. Total small intestine (Table 4) or small intestine divided up into 3 equally long pieces (Table 5) were analysed for cryptdin and CRS content in different mouse strains using qRT-PCR. NMRI/KI, C3H/HeN and FVB strains share a similar expression profile, while C57BL/6 clearly diverges. When comparing cryptdin 4 expression, it is clear that C57BL/6, which harbours a unique cryptdin 4 variant, had the highest level with on average 30-fold higher expression (6 × 10^6 ^copies vs. average of 2 × 10^5 ^copies). Cryptdin 4 from C57BL/6 also increased from duodenum to ileum (Table 5). C57BL/6 mice do not express CRS peptides detectable with the primers used for CRS4C. Within the other strains of mice the levels of the different products do not vary more than three-fold.

**Table 4:** Differences in mRNA expression of CRS and cryptdins in small intestine of different mouse strains

	**C57BL/6, N = 2**	**NMRI/KI, N = 4**	**C3H/HeN, N = 4**
**CRS1C**	2.0 × 10^6^	7.5 × 10^5^	1.1 × 10^6^
**CRS4C**	Not present	3.0 × 10^7^	1.4 × 10^6^
**Crypt1**	4.4 × 10^6^	5.6 × 10^6^	6.9 × 10^6^
**Crypt4**	Not present	2.3 × 10^5^	1.9 × 10^5^
**Crypt4 C57BL/6**	5.8 × 10^6^	Not present	Not present

Average mRNA levels in whole small intestine of adult C57BL/6, NMRI/KI and C3H/HeN mice measured by qRT-PCR. N = number of mice. Numbers denotes mRNA copy numbers per 10 ng RNA. Note that C57BL/6 mice lack CRS4C-1 to 3 and cryptdin 4 and display a unique cryptdin 4 variant.

**Table 5:** Regional differences in mRNA expression of CRS and cryptdins in small intestine of different mouse strains

	**C57BL/6**	**FVB**
**CRS1C**	D = 1.8 × 10^6^	D = 7.6 × 10^5^
	J = 2.6 × 10^6^	J = 4.3 × 10^5^
	I = 2.5 × 10^6^	I = 1.1 × 10^5^
		
**CRS4C**	Not present	D = 1.2 × 10^5^
		J = 1.2 × 10^7^
		I = 2.5 × 10^7^
		
**Crypt1**	D = 2.2 × 10^6^	D = 3.3 × 10^6^
	J = 3.0 × 10^6^	J = 3.2 × 10^6^
	I = 5.6 × 10^6^	I = 2.2 × 10^6^
		
**Crypt4**	Not present	D = 1.6 × 10^4^
		J = 1.1 × 10^5^
		I = 2.5 × 10^5^
		
**Crypt4 C57BL/6**	D = 1.4 × 10^5^	Not present
	J = 4.7 × 10^6^	
	I = 8.7 × 10^6^	

Expression of CRS and cryptdin in different parts of the small intestine measured by qRT-PCR. Tissues pooled from three mice. D = duodenum, J = jejunum, I = ileum. Numbers denotes mRNA copy numbers per 10 ng RNA. Note that C57BL/6 mice lack CRS4C-1 to 3 and cryptdin 4 and display a unique cryptdin 4 variant.

## Discussion

The small intestine is a heterogeneous tissue with longitudinal differences in nutrient adsorption capacity and in resident microbiota. The bias in colonization of the terminal small intestine depends on host factors rather than initial bacterial input [18]. Here we show that Paneth cell derived antimicrobial peptides differ along the length of the small intestine, and may be one factor that contributes to colonization. Other host factors that may influence colonization include pH, peristalsis and glycans [19]. Based on human association studies [12] and transgenic mice [13,14], it is clear that antimicrobial peptides in the small intestine play important functions both in man and mouse. The characteristic localization in the small intestine of celiac and Crohn's diseases to the duodenum and ileum, respectively, might be linked to differences in innate functions and regional expression/release of defensins.

Two families of defensins/defensin-like antibacterial molecules are expressed in Paneth cells of mouse small intestine: cryptdins and CRS4C, whereas information is lacking on the antibacterial properties of CRS1C. Cryptdins, with the exception of cryptdin 4 [16,17], and CRS1C are equally expressed along the small intestine, and represent "class I" defensins. CRS4C and cryptdin 4, representing "class II" defensins, are shown to be more restricted to the ileal region of the small intestine. Together with other antimicrobial factors this likely creates a distinct antimicrobial milieu in the ileum as compared to duodenum. Similarly, the human enteric α-defensin 5 and 6 also increase along the small intestinal axis [20] without a concomitant increase in Paneth cell numbers [21]. Patil and co-workers [15] showed that also rat antimicrobial peptide genes expressed in small intestine corresponded to two groups with one expressed in equal numbers along the intestinal axis while the other group increased.

The two proteins with most prominent differences in expression between germ-free and colonized mice, CRS4C (this study) and RegIIIγ [4] both have important longitudinal differences in intestinal expression. Expression of sPLA2 also varied between germ-free and colonized conditions. Cryptdins are constitutively expressed, even under germ-free conditions ([22] and this study). While this study was under review, Inoue *et al *published a detailed study on the developmental expression of cryptdin 1–6 in germ-free and conventional BALB/c mice, showing enhanced expression in conventional mice of all cryptdins, especially after weaning [23]. The bacteria do not appear to influence the activation of cryptdin peptides since germ-free mice produce mature cryptdins [22]. Thus, much of the peptide repertoire is formed independently of colonization, although we in general detected Paneth cell products at lower levels in germ-free mice, possibly correlating with decreased Paneth cell numbers [24]. We did not observe any altered synthesis of CRS peptides (or cryptdins) in adult mice after oral challenge with the pathogenic bacteria Salmonella or Listeria. Cryptdin expression has been reported to be repressed in FVB mice after wild type Salmonella infection with maximum decrease after 18 hours [25]. Our results are generated in C3H/HeN mice and analysed for up to 8 hours. The models are therefore not directly comparable. It is possible that a response to invasive pathogens can occur at the processing or granule release level rather than increased synthesis, which may already be at high capacity.

Paneth cell linage emerges in mouse foetal small intestine with characteristic morphology of Paneth cells (including granules) on post-natal day 7 (P7). The lineage then expands greatly during the period of days P14–P21 [26,27]. Here we show that the expression levels of CRS and other antimicrobial peptides are detectable at day 14, but to a different degree for individual products. The differentiation of intestinal stem cells and commitment to the Paneth cell lineage is dependent on several transcription factors (reviewed in [28]). The transcription factor TCF-4 plays a major role in both early differentiation and in the maturation of Paneth cells [8] following Wnt/β-catenin mediated signalling [29]. TCF-4 also regulates the expression of class I defensins, including cryptdin 1 [7,8], CRS1C [30], as well as α-defensin 5 and 6 in humans [7,8]. We find it more probable, however, that additional/other transcription factors, with a gradually increasing expression pattern throughout the small intestine regulates class II defensins such as CRS4C. One putative transcription factor could be the newly described intestine-specific homeobox; Isx [31]. Isx has a selective expression in the small intestine with an increase from duodenum to ileum. Two other putative transcription factors are ME1 and PPARβ [32,33].

Cryptdin and CRS genes in mice are located adjacently on chromosome 8, in a region known to have undergone significant changes through evolution [15]. The precise number of genes is not clear, but a large number of different CRS mRNA sequences has been identified in various mouse strains, including 129/SVJ (8), C3H/HeN (17) and in FVB mice (13) ([6,11], this study) that generates striking differences in expression pattern. For example, CRS4C is the dominant peptide expressed by C3H/HeN, NMRI/KI and FVB mice but C57BL/6 mice lack this peptide family and perhaps compensate by expressing higher amounts of the C57BL/6 specific cryptdin 4. The variation in peptide repertoire may very well be responsible for a difference in microbiota between mice. This idea is strengthened by experiments with transgenic mice in which Paneth cell expression of a human counterpart of cryptdins, α-defensin 5, changed the composition of the flora [12]. If variations in peptide-mediated defense between mouse strains confer differences in intestinal microbiota, then it is an open question to whether this can be one of the explanations for the difference in susceptibility to oral infection.

## Conclusion

The repertoire of mouse Paneth cell antimicrobial molecules shows significant regional variation between duodenum and ileum. Expression of some of these effector molecules was up-regulated in conventional versus germ-free mice, but the majority did not change. Together, these observations indicate that multiple regulatory pathways coordinate the expression of Paneth cell antimicrobial molecules. The overall repertoire may play an important role in local bacteria-host homeostasis, in addition to their proposed role in protection against infection.

## Methods

### Mouse strains and tissues

C3H/HeN, NMRI/KI, FVB, BALB/c and C57BL/6 adult mice were housed under standard pathogen-free conditions. Adult mice were defined as 7 weeks for Figure 1, 4–6 weeks for Figure 2A–B and 3A–B, 3 weeks for Figure 2C and 5B, 8–12 weeks for Figure 4 and 6–12 weeks for Figure 5A. Germ-free NMRI/KI mice were housed in sterile environment. The designation "duodenum" represents the proximal 1/3 of the small intestine, whereas "jejunum" and "ileum" were the medial 1/3 and the distal 1/3, respectively. In qRT-PCR assays of small intestine of FVB mice, six cm of the most proximal, most distal and the medial part were used. Eight centimetres of proximal and eight centimetres of distal small intestine were excised from 14 and 18 day old FVB mice. Large intestine was divided into three equally sized pieces and named ascending-trans- and descending colon. All tissues used for RNA isolation except for small intestine were collected and pooled from several animals. Small intestine, caecum and thymus from C3H/HeN mice were used for Western blot and immunohistochemistry.

### Mouse infections

Three female C3H/HeN mice were intragastrically infected with 8 × 10^8 ^*Salmonella enterica *serovar typhimurium strain ATCC 14028 and whole small intestine was removed at 30 min, 2 h or 8 h post infection. Control mice received phosphate buffered saline. Three BALB/c female mice were orally challenged with 10^9 ^*Listeria monocytogenes *type 1 clinical isolate and small intestine was removed after three days. Control mice received phosphate buffered saline, and livers and spleens were homogenized and plated to access infection status.

### Antibodies and peptides

Synthetic CRS4C-3 (LGWGRRCPRCPPCPNCRRCPRCPTCPSCNCNPK) and cryptdin 2 (LRDLVCYCRTRGCKRRERMNGTCRKGHLMYTLCCR) peptides were used to raise antibodies in rabbits (Innovagen, Lund, Sweden). The antibodies were purified using protein G. The synthetic peptides were used as standards for Western blot.

### RNA preparation and reverse transcriptase PCR

Tissue was snap frozen in liquid nitrogen or alternatively placed in RNAlater (Ambion, Austin, TX, USA) for storage and RNA was subsequently isolated with guanidine isothiocyanate buffer followed by a 5.7 M cesium chloride cushion [34]. Five micrograms of RNA were used with Oligo-(dT)_12–18 _for reverse-transcriptase PCR with GIBCO BRL superscript first strand synthesis system (Grand Island, NY, USA) in accordance to the manufacturer's recommendations. The polyA specific adaptor primer 5'-ttctagaattcagcggccgc(t)_30_NN-3' was used for reverse transcriptase PCR of CRS1C and CRS4C. The cDNA was purified with Qiaquick PCR purification kit (Qiagen, Hilden, Germany).

### Cloning of CRS and other antimicrobial peptide products

Cloning of products was performed with standard procedures [20]. Briefly, 10–100 ng of cDNA was amplified with gene-specific primers with Roche FastStart Taq DNA Polymerase (Roche Applied Science, Mannheim, Germany). The product was cloned into pBluescript II and transfected into DH5α competent cells (both from Invitrogen, Carlsbad, CA, USA). Plasmid was isolated, sequenced, quantified and diluted in series of 10-fold dilutions in 0.02 g/l torula yeast RNA (Sigma-Aldrich, St Louis, MO, USA). The procedures for cloning of CRS sequences were the same as above except for two different forward primers and an adaptor specific reverse primer in the initial step (Table 1).

### Quantitative reverse transcriptase PCR

Roche Lightcycler with Lightcycler-FastStart DNA Master SYBR Green I kit (Roche) was used to make standard series of each cloned product with its matched primers, Table 1. The standard was used to measure gene copy number of the unknown sample derived from 10 ng of RNA as previously described [20]. Samples were measured in duplicates. The CRS4C assay did not cross-react with CRS1C and vice versa. Gene copy numbers below 100 copies are reported as absent.

### Immunohistochemistry

Ileum from a C3H/HeN mouse was fixed with formalin (Histolab, Göteborg, Sweden). After deparafinization, slides were submerged in boiling antigen retrieval buffer (1 mM sodium citrate, 1 mM citric acid in water pH 6) for two minutes. Cells were permeabilized with 0.1% triton-X100 before incubation with CRS4C antibody. FITC conjugated AffiniPure F(ab')2 fragment of donkey anti-rabbit IgG (H+L) antibodies were used for detection (Jackson, West Grove, PA, USA). Samples were washed three times in phosphate buffered saline (PBS) in between each incubation step. Hoechst 33258 (Sigma-Aldrich) staining was used to visualize the nucleus.

### Protein extraction from mouse tissue

Tissues were ground in liquid nitrogen and extracted (one hour, +4°C) in final volumes of 60% acetonitrile and 1% triflouroacetic acid. The homogenate was clarified with centrifugation, the supernatant was lyophilised, and re-dissolved in the same volume of 20% ethanol as the ground tissue weight, as described in [22].

### Western blot

Protein extracts were mixed with 4 M urea (final concentration), NuPAGE LDS Sample buffer (Invitrogen) and 10% β-mercaptoethanol before heating at 100°C. Iodoacetamide (final concentration 50 mM) was added and extract originating from 2.5 μg tissue was loaded into each lane. Proteins were separated using 1.0 mm 4–12% NuPAGE Bis-Tris gels in NuPAGE MES SDS running buffer and blotted onto polyvinylidene difluoride filters (all from Invitrogen). The filters were blocked in 2% dried milk in PBS with 0,1% Tween (PBST). Incubation with antigen-specific antibodies and horseradish peroxidase (HRP)-conjugated anti-rabbit antibody were in 1% bovine serum albumin in PBST. Bound antibody was detected by chemiluminescence (SuperSignal West Dura, Pierce Biotechnology, Rockford, IL, USA). Densitometric readings of the blots were accessed with Image gauge v4 (Fujifilm, Stamford, CT, USA). Three different dilutions of synthetic CRS4C-3 and cryptdin 2 peptide were analysed simultaneously and protein bands were quantified.

### Statistical analyses

Error bars in graphs represent range. Significance of 95% was evaluated with Mann-Whitney two tailed test.

## Abbreviations

qRT-PCR: quantitative real-time reverse transcriptase-polymerase chain reaction; CRS: cryptdin-related sequences; Crypt1: cryptdin 1 family of sequences; Crypt4: cryptdin 4; mLys: myeloid-specific lysozyme; pLys: Paneth cell specific lysozyme; sPLA2: secretory phospholipase A2 type IIa; GAPDH: glyceraldehyde-3-phosphate dehydrogenase; TCF-4: transcription factor 7-like 2, also known as TCF7L2; NOD2: nucleotide-binding oligomerization domain containing 2; RegIIIγ: regenerating islet-derived 3 gamma; MMP7: matrix metallopeptidase 7/matrilysin.

## Authors' contributions

JK designed and cloned the transcripts used for qRT-PCR of CRS, isolated and quantified RNA transcripts except for TCF-4, aligned and compared the different CRS transcripts, carried out the immunoassays, performed the statistical analyses, and drafted the manuscript. HC and RJK designed and cloned the transcripts used for qRT-PCR except for CRS and lysozyme, and measured transcript levels of TCF-4. JK, KP, CLB and MA participated in the design and coordination and helped to finalize the manuscript. All authors read and approved the final manuscript.

## References

[B1] Ireland H, Houghton C, Howard L, Winton DJ (2005). Cellular inheritance of a Cre-activated reporter gene to determine Paneth cell longevity in the murine small intestine. Dev Dyn.

[B2] Ayabe T, Satchell DP, Wilson CL, Parks WC, Selsted ME, Ouellette AJ (2000). Secretion of microbicidal alpha-defensins by intestinal Paneth cells in response to bacteria. Nat Immunol.

[B3] Ouellette AJ, Selsted ME (1996). Paneth cell defensins: endogenous peptide components of intestinal host defense. Faseb J.

[B4] Cash HL, Whitham CV, Behrendt CL, Hooper LV (2006). Symbiotic bacteria direct expression of an intestinal bactericidal lectin. Science.

[B5] Jones DE, Bevins CL (1993). Defensin-6 mRNA in human Paneth cells: implications for antimicrobial peptides in host defense of the human bowel. FEBS Lett.

[B6] Huttner KM, Ouellette AJ (1994). A family of defensin-like genes codes for diverse cysteine-rich peptides in mouse Paneth cells. Genomics.

[B7] Andreu P, Colnot S, Godard C, Gad S, Chafey P, Niwa-Kawakita M, Laurent-Puig P, Kahn A, Robine S, Perret C (2005). Crypt-restricted proliferation and commitment to the Paneth cell lineage following Apc loss in the mouse intestine. Development.

[B8] van Es JH, Jay P, Gregorieff A, van Gijn ME, Jonkheer S, Hatzis P, Thiele A, Born M van den, Begthel H, Brabletz T (2005). Wnt signalling induces maturation of Paneth cells in intestinal crypts. Nat Cell Biol.

[B9] Ouellette AJ, Lualdi JC (1990). A novel mouse gene family coding for cationic, cysteine-rich peptides. Regulation in small intestine and cells of myeloid origin. J Biol Chem.

[B10] Lin MY, Munshi IA, Ouellette AJ (1992). The defensin-related murine CRS1C gene: expression in Paneth cells and linkage to Defcr, the cryptdin locus. Genomics.

[B11] Hornef MW, Putsep K, Karlsson J, Refai E, Andersson M (2004). Increased diversity of intestinal antimicrobial peptides by covalent dimer formation. Nat Immunol.

[B12] Wehkamp J, Salzman NH, Porter E, Nuding S, Weichenthal M, Petras RE, Shen B, Schaeffeler E, Schwab M, Linzmeier R (2005). Reduced Paneth cell alpha-defensins in ileal Crohn's disease. Proc Natl Acad Sci USA.

[B13] Wilson CL, Ouellette AJ, Satchell DP, Ayabe T, Lopez-Boado YS, Stratman JL, Hultgren SJ, Matrisian LM, Parks WC (1999). Regulation of intestinal alpha-defensin activation by the metalloproteinase matrilysin in innate host defense. Science.

[B14] Salzman NH, Ghosh D, Huttner KM, Paterson Y, Bevins CL (2003). Protection against enteric salmonellosis in transgenic mice expressing a human intestinal defensin. Nature.

[B15] Patil A, Hughes AL, Zhang G (2004). Rapid evolution and diversification of mammalian alpha-defensins as revealed by comparative analysis of rodent and primate genes. Physiol Genomics.

[B16] Darmoul D, Ouellette AJ (1996). Positional specificity of defensin gene expression reveals Paneth cell heterogeneity in mouse small intestine. Am J Physiol.

[B17] Ouellette AJ, Darmoul D, Tran D, Huttner KM, Yuan J, Selsted ME (1999). Peptide localization and gene structure of cryptdin 4, a differentially expressed mouse paneth cell alpha-defensin. Infect Immun.

[B18] Rawls JF, Mahowald MA, Ley RE, Gordon JI (2006). Reciprocal gut microbiota transplants from zebrafish and mice to germ-free recipients reveal host habitat selection. Cell.

[B19] Hao WL, Lee YK (2004). Microflora of the gastrointestinal tract: a review. Methods Mol Biol.

[B20] Wehkamp J, Chu H, Shen B, Feathers RW, Kays RJ, Lee SK, Bevins CL (2006). Paneth cell antimicrobial peptides: Topographical distribution and quantification in human gastrointestinal tissues. FEBS Lett.

[B21] Kelly P, Feakins R, Domizio P, Murphy J, Bevins C, Wilson J, McPhail G, Poulsom R, Dhaliwal W (2004). Paneth cell granule depletion in the human small intestine under infective and nutritional stress. Clin Exp Immunol.

[B22] Putsep K, Axelsson LG, Boman A, Midtvedt T, Normark S, Boman HG, Andersson M (2000). Germ-free and colonized mice generate the same products from enteric prodefensins. J Biol Chem.

[B23] Inoue R, Tsuruta T, Nojima I, Nakayama K, Tsukahara T, Yajima T (2008). Postnatal changes in the expression of genes for cryptdins 1–6 and the role of luminal bacteria in cryptdin gene expression in mouse small intestine. FEMS Immunol Med Microbiol.

[B24] Satoh Y (1988). Effect of live and heat-killed bacteria on the secretory activity of Paneth cells in germ-free mice. Cell Tissue Res.

[B25] Salzman NH, Chou MM, de Jong H, Liu L, Porter EM, Paterson Y (2003). Enteric salmonella infection inhibits Paneth cell antimicrobial peptide expression. Infect Immun.

[B26] Bry L, Falk P, Huttner K, Ouellette A, Midtvedt T, Gordon JI (1994). Paneth cell differentiation in the developing intestine of normal and transgenic mice. Proc Natl Acad Sci USA.

[B27] Darmoul D, Brown D, Selsted ME, Ouellette AJ (1997). Cryptdin gene expression in developing mouse small intestine. Am J Physiol.

[B28] Hauck AL, Swanson KS, Kenis PJ, Leckband DE, Gaskins HR, Schook LB (2005). Twists and turns in the development and maintenance of the mammalian small intestine epithelium. Birth Defects Res C Embryo Today.

[B29] Korinek V, Barker N, Morin PJ, van Wichen D, de Weger R, Kinzler KW, Vogelstein B, Clevers H (1997). Constitutive transcriptional activation by a beta-catenin-Tcf complex in APC-/- colon carcinoma. Science.

[B30] Wehkamp J, Wang G, Kubler I, Nuding S, Gregorieff A, Schnabel A, Kays RJ, Fellermann K, Burk O, Schwab M (2007). The Paneth cell alpha-defensin deficiency of ileal Crohn's disease is linked to Wnt/Tcf-4. J Immunol.

[B31] Choi MY, Romer AI, Hu M, Lepourcelet M, Mechoor A, Yesilaltay A, Krieger M, Gray PA, Shivdasani RA (2006). A dynamic expression survey identifies transcription factors relevant in mouse digestive tract development. Development.

[B32] Tanigawa Y, Yakura R, Komiya T (2007). The bHLH transcription factor Tcf12 (ME1) mRNA is abundantly expressed in Paneth cells of mouse intestine. Gene Expr Patterns.

[B33] Varnat F, Heggeler BB, Grisel P, Boucard N, Corthesy-Theulaz I, Wahli W, Desvergne B (2006). PPARbeta/delta regulates paneth cell differentiation via controlling the hedgehog signaling pathway. Gastroenterology.

[B34] Chirgwin JM, Przybyla AE, MacDonald RJ, Rutter WJ (1979). Isolation of biologically active ribonucleic acid from sources enriched in ribonuclease. Biochemistry.

